# *Magnolia kobus* Extract Inhibits Periodontitis-Inducing Mediators in *Porphyromonas gingivalis* Lipopolysaccharide-Activated RAW 264.7 Cells

**DOI:** 10.3390/cimb45010036

**Published:** 2023-01-06

**Authors:** Hae-Jin Lee, So-Jung Lee, Sung-Kwon Lee, Bong-Keun Choi, Dong-Ryung Lee

**Affiliations:** Research Institute, NUON Co., Ltd., Seongnam 13201, Republic of Korea

**Keywords:** *Magnolia kobus*, magnolin, RAW 264.7, periodontitis, *Porphyromonas gingivalis*, inflammation, MMPs, NF-κB, MAPK

## Abstract

Periodontitis, a disease caused by inflammation of oral bacteria, contributes to the loss of alveolar bone and destruction of connective tissues. *Porphyromonas gingivalis*, a Gram-negative bacterium, is known to possess important pathogenic factors for periodontal disease. In this study, we investigated the anti-periodontitis effects of *Magnolia kobus* extract (MKE) and magnolin as a component of *Magnolia kobus* (MK) in murine macrophage RAW 264.7 cells stimulated with *Porphyromonas gingivalis* lipopolysaccharide (LPS). Effects of MKE and magnolin on the mechanism of RAW 264.7 cellular inflammation were determined by analyzing nitric oxide (NO) production and Western blot protein expression (*n* = 3). MKE/magnolin inhibited NO production without affecting cell survival. MKE/magnolin treatment inhibited LPS-induced pro-inflammatory cytokines, expression levels of matrix metalloproteinases (MMPs such as MMP-1, 3, 8, 9, and 13), and protein levels of inflammatory mediators (such as TNF-α, IL-1β, and mPGES-1). MKE/magnolin also suppressed NF-κB activation by inhibiting the TLR4 signaling pathway. These findings suggest that MKE has a therapeutic effect on inflammatory periodontal disease caused by oral bacterium *P. gingivalis* and that magnolin is a major functional component in the anti-inflammatory effect of MKE.

## 1. Introduction

Periodontitis is a common chronic inflammatory disease that includes gingival inflammatory response, connective tissue loss, and weakened alveolar bones that support roots of teeth, leading to tooth loss [1]. In periodontal disease, the inflammatory process is the organism’s response to microbial biofilm formation and results in collateral tissue damage [2]. Periodontal diseases are very prevalent, affecting more than 90% of the world’s population. It has been reported that approximately 22% of US adults have a mild periodontal disease and 13% have a moderate or severe periodontal disease [3]. Recently, the WHO has argued that the importance of global periodontal disease control should be strengthened [4].

A common, mild form of gingivitis causes irritation, redness, and swelling of the gum due to an inflammatory reaction. This continuative inflammatory response can cause irreversible periodontal diseases and structure changes such as the loss of teeth, which can be related to the development of chronic diseases such as cardiovascular disease, diabetes, metabolic abnormalities, and cancer [5,6,7].

*Porphyromonas gingivalis*, one of the representative periodontal disease pathogens in oral bacteria, can produce lipopolysaccharide (LPS) endotoxins that can stimulate the induction of inflammatory cytokines and contribute to the process of alveolar bone loss and connective tissue destruction [8,9,10]. In addition, the secretion of various inflammatory cytokines including tumor necrosis factor-alpha (TNF-α) generated by periodontal pathogen stimulation can act as a major factor in periodontal tissue destruction [11,12]. In particular, TNF-α is a factor that can induce osteoclast differentiation in macrophages. It can recruit TNF receptor-associated factors (TRAFs) to sequentially activate transcriptional factors nuclear factor kappa B (NF-κB) [13,14].

Activation of macrophages can cause the release of extracellular matrix metalloproteinases (MMPs) responsible for periodontal tissue destruction and connective tissue damage [15]. MMPs secreted by stimulation of cytokines produced by periodontal pathogens can cause gingival degeneration by decomposing collagen, a periodontal tissue. MMP-1 (collagenase-1) is an enzyme synthesized and secreted by macrophages, connective tissue cells, chondroblasts, keratinocytes, endothelial cells, and osteoblasts. MMP-8 (collagenase-2) is packed in specific granules of neutrophil granulocytes [16,17]. MMP-1 and MMP-8 are representative enzymes known to destroy periodontal tissues, both of which are responsible for the breakdown of collagen types I, II, and III [18,19,20]. As a collagenase, MMP-13 is considered to play an important role in periodontal matrix degradation [21]. MMP-9 belongs to gelatinases, which are mainly secreted by progressive multifocal leukoencephalopathy that can degrade collagen type IV present in gingival tissues [22,23]. Stromelysin-1 (MMP-3) is a pivotal activator that can activate latent MMPs including pro-MMP-1, -8, and -9. It can also degrade proteoglycan and fibronectin as a proteolytic enzyme, destroy connective tissues, and accelerate periodontitis [24,25]. Therefore, the regulation of inflammatory cytokines and MMPs might play an important role in preventing the progression of periodontal diseases.

**Figure 1:**
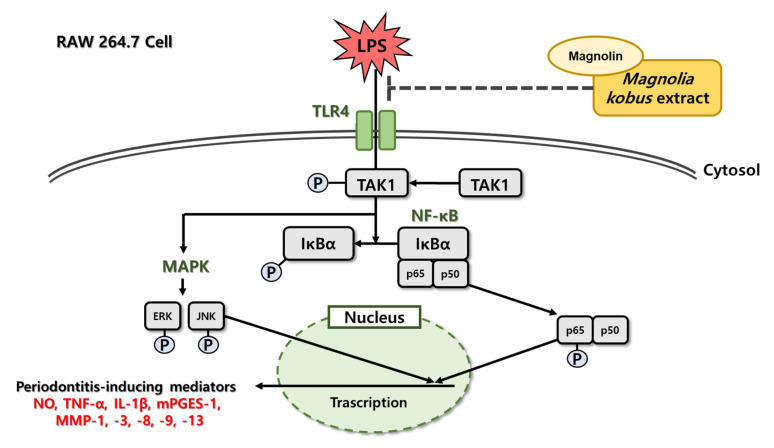
The anti-inflammatory pathway of MKE in RAW 264.7 cells.

*Magnolia kobus* (MK) is a flowering plant native to tropical America and the subtropics in Asia (Korea, China and Japan). Its parts such as bark, flowers, and fruits are used in traditional medicine to treat chronic rhinitis and colds [26]. In previous studies on *Magnoliae* genus, a *Streptococcus mutans-*inhibiting effect of *Magnolia grandiflora* [27], anti-inflammatory activity of magnolol isolated from *Magnoliae cortex* [28], and inflammatory response-controlling effect of *Manoliae cortex* and *zea mays* L complex extract have been reported [29]. Magnolin, an active lignan present in *Magnolia*, has various effects such as anti-cancer, anti-inflammatory, and antioxidant effects [30,31,32]. In addition, it has been reported that magnolin can suppress the activation of NF-κB pathway with significant anti-inflammatory physiological activity in chondrocytes to alleviate osteoarthritis [33]. However, as to whether MK could improve periodontal disease caused by *P. gingivalis* and its mechanism of action remains unclear. In order to investigate the anti-periodontitis effects of MK, macrophages were treated with MK extract (MKE) and its active compound, magnolin, and the inflammatory responses were evaluated (Figure 1).

Thus, the purpose of this study was to determine whether MKE and its biologically active compound, magnolin, could be used to treat periodontal diseases through its anti-inflammatory effects and MMPs inhibitory ability in *P. gingivalis* LPS-stimulated macrophage RAW 264.7 cells.

## 2. Materials and Methods

### 2.1. Materials

Magnolin was supplied by Sigma-Aldrich (St. Louis, MO, USA). Primary antibodies against IL-1β was obtained from the Invitrogen Life Technologies (Carlsbad, CA, USA). Anti-MMP-3, -8, -9, -13, mPGES-1, and TNF-α antibodies were purchased from Abcam Inc. (Cambridge, CA, USA). Anti-MMP-1, pTAK1, phospho-ERK, ERK, phospho-JNK, JNK, NF-κB pathway antibody sampler kit and β-actin were purchased from Cell Signaling Technology (Danvers, MA, USA). Goat anti-Mouse lgG-HRP and Goat anti-Rabbit lgG-HRP were purchased from GenDEPOT (Barker, TX, USA).

### 2.2. Preparation of Magnolia kobus Extract

*Magnolia kobus* extract was supplied by NUON Co., Ltd., (Seongnam, Korea). MK was dried and extracted by aqueous water. The solution was filtered, concentrated, and dried to obtain MK extract. The magnolin content was determined at approximately 1.5% in MKE by quantitative analysis (Data not shown).

### 2.3. Cell Culture

The murine macrophage RAW 264.7 cells (American Type Culture Collection, Rockville, MD, USA) were cultured in Dulbecco’s modified minimal essential medium (DMEM; Gibco, Grand Island, NY, USA) with 10% fetal bovine serum (FBS; Gibco, Grand Island, NY, USA) and 1% penicillin–streptomycin (Gibco, Grand Island, NY, USA) at 37 °C cell incubator humified atmosphere with 5% CO2. MKE and magnolin were dissolved in dimethyl sulfoxide (DMSO; Wako, Osaka, Japan). Samples dissolved in DMSO were diluted 1/1000 in the medium and treated with cells.

### 2.4. Cell Viability Assay

A 3-(4,5-dimethylthiazol-2-yl)-2,5-diphenyl tetrazolium bromide (MTT; Duchefa Biochemie, Haarlem, Netherlands) assay was performed to determine the cytotoxicity of MKE and magnolin to RAW 264.7 cells. Cells were treated with MKE (30, 100 or 300 μg/mL) and magnolin (0.45, 1.5 or 4.5 μg/mL) for 24 h. MTT solution was added to each well to a final concentration of 0.5 mg/mL, and then the plate was incubated at 37 °C for 2 h. After 2 h, the supernatant was removed, and 100 μL DMSO was added to each well to dissolve formazan crystals. The dissolved formazan was measured at a wavelength of 570 nm in a microplate plate reader (Tecan, Mannedorf, Switzerland).

### 2.5. Nitric Oxide (NO) Assay

RAW 264.7 cells were seeded into a 48-well microplate at a density of 2.0 × 10^4^ cells/well. After 24 h, cells were pretreated for 2 h with MKE and magnolin at concentrations that were confirmed to be nontoxic in a 24 h cell viability assay. RAW 264.7 cells were stimulated with lipopolysaccharide from *Porphyromonas gingivalis* (Sigma-Aldrich, MO, USA) (100 ng/mL), as well as MKE (30, 100 or 300 μg/mL) and magnolin (0.45, 1.5 or 4.5 μg/mL) for 24 h, then the cell culture supernatants were harvested and briefly centrifuged. For NO concentration, 50 µL of cell culture supernatant was harvested and mixed with 100 µL of Griess reagent (Promega, Madison, WI, USA) according to the manufacturer’s protocol. The mixture was reacted for 10 min under dark conditions and absorbance was determined using a microplate reader at a wavelength of 425 nm.

### 2.6. Protein Extraction and Western Blot Analysis

After removing the culture media of macrophage RAW 264.7 cells, the cells were scraped with CelLytic buffer (Sigma-Aldrich, MO, USA). The cell lysate was centrifuged at 13,000 rpm at 4 °C for 15 min. The protein concentration of the cell lysate supernatant was quantified by the Bradford assay (Bio-Rad Laboratories, Hercules, CA, USA), and all samples were diluted with CelLytic buffer to have the same concentration of protein (20 μg/20 μL). Proteins were separated by sodium dodecyl sulfate polyacrylamide gel electrophoresis (SDS-PAGE). SDS-gel was transferred to an Immobilon-P membrane (Millipore, Bedford, MA, USA) and blocked with 5% skim milk for 30 min. After blocking, the membrane was incubated with specific primary antibodies at 4 °C overnight. Blots were washed three times with Tris-buffered saline with 0.5% Tween 20 (TBS-T) and then incubated with corresponding horseradish peroxidase-conjugated anti-mouse or anti-rabbit immunoglobulin G for 1 h. ECL solution (GenDEPOT, Barker, TX, USA) was dispensed on the membrane, and protein bands were measured using LuminoGraph (Atto, Tokyo, Japan). Band images and intensities were quantified in the ImageJ program (NIH; Bethesda, MD, USA) and corrected by β-actin levels.

### 2.7. Statistical Analysis

In this study, all data are expressed as mean ± standard deviation. All statistical analyses were performed using SPSS version 12.0.0 (IBM Co., Armonk, NY, USA). Normality of the distribution of the results was verified using the Shapiro–Wilk test, and the homogeneity of variances using the Levene’s test. The normal data (*p* > 0.05) continued to be analyzed. Significance between the LPS-activated control group and vehicle control group was determined using the Student’s *t*-test. Significance between MKE/magnolin treatment group and LPS activated control group was determined using the Student’s *t*-test. Significant difference was considered at *p* < 0.05. All experiments were performed in triplicates.

## 3. Results

### 3.1. Cytotoxicity of MKE/Magnolin to RAW 264.7 Cells

**Figure 2:**
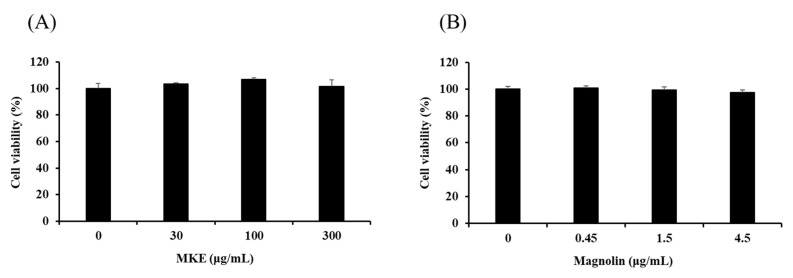
Effect of (**A**) MKE (0, 30, 100, and 300 µg/mL) and (**B**) magnolin (0, 0.45, 1.5, and 4.5 µg/mL) on RAW 264.7 cell viability. MKE and magnolin were used for 24 h of treatment. Values are represented as means ± standard deviation.

Before evaluating the in vitro efficacy of MKE/magnolin in RAW 264.7 cells, effects of MKE/magnolin at different concentrations on cell viability were tested (Figure 2). Cells were treated with MKE at 30, 100, or 300 μg/mL for 24 h. Cell viability was then measured by the MTT assay. MKE at concentrations up to 300 μg/mL did not show cytotoxicity, indicating that subsequent experimental results were not due to decreased cell viability. According to the content of magnolin in MKE, cells were treated with magnolin at 0.45, 1.5, and 4.5 μg/mL for 24 h. Magnolin did not show cytotoxicity at these concentrations.

### 3.2. Effects of MKE/Magnolin on NO Production by RAW 264.7 Cells

**Figure 3:**
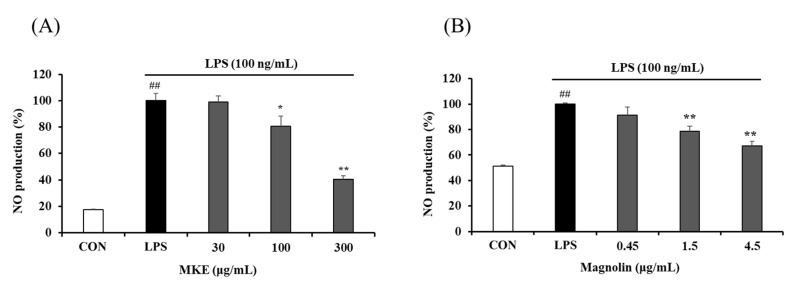
Effect of (**A**) MKE (30, 100, and 300 µg/mL) and (**B**) magnolin (0.45, 1.5, and 4.5 µg/mL) on RAW 264.7 cell NO production. MKE and magnolin were used for 24 h with LPS. Nitrite formed in the supernatant was measured using Griess’ reagent. Values represent means ± standard deviation. *## p* < 0.01 vs. Vehicle control group; * p < 0.05 and ** p < 0.01 vs. LPS-activated control group.

LPS-induced RAW 264.7 cells showed a significant increase (*## p* < 0.01) compared to LPS-untreated cells (vehicle-treated control cells). On the other hand, MKE treatment inhibited NO production in a dose-dependent manner, with inhibition rates of 19.20 (±3.28)% and 59.43 (±1.03)% at 100 and 300 μg/mL, respectively (** *p* < 0.01). Magnolin at concentrations of 1.5 and 4.5 μg/mL also resulted in 21.26 (±3.90)% and 32.87 (±3.60)% reductions of NO production, respectively, compared to the control group (LPS treatment alone) (** p < 0.01) (Figure 3).

### 3.3. MKE/Magnolin Inhibits Production of Inflammatory Mediators

**Figure 4:**
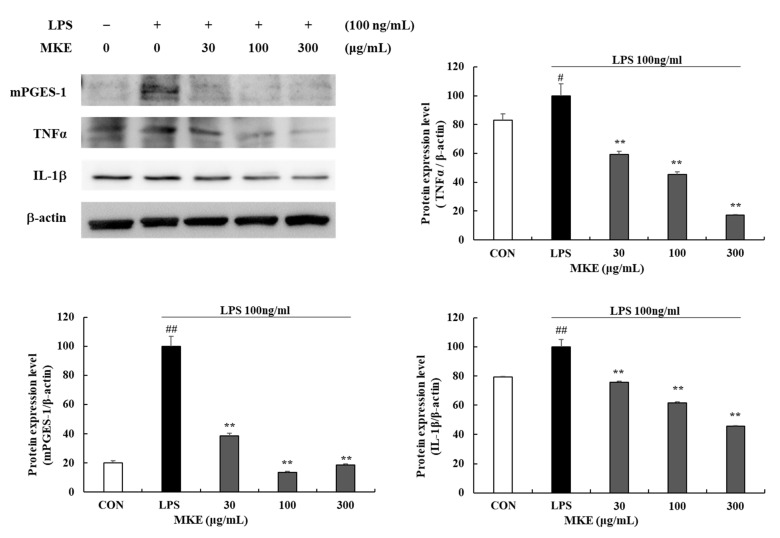
Effects of MKE (30, 100, and 300 µg/mL) on the production of inflammatory mediators in LPS-stimulated RAW 264.7 cells. MKE and 100 ng/mL of LPS was added except for the vehicle control group and incubated at 37 °C for 24 h. Protein expression levels of TNF-α, IL-1β and mPGES-1 were detected by Western blot. The protein band density was measured by utilizing the ImageJ software. β-actin was used as a control. Values represent means ± standard deviation. *# p* < 0.05 and *## p* < 0.01 vs. Vehicle control group; ** p < 0.01 vs. LPS-activated control group.

LPS-induced RAW 264.7 cells showed significant *(# p* < 0.05, *## p* < 0.01) increases of inflammatory mediators such as microsomal prostaglandin E synthase-1 (mPGES-1), TNF-α, and interleukin (IL)-1β (Figure 4). MKE treatment significantly inhibited protein expression levels of mPGES-1, TNF-α, and IL-1β inflammatory mediators. In addition, TNF-α protein expression levels were reduced by 40.70 (±2.05)%, 54.45 (±1.53)% and 82.83 (±0.50)% in cells treated with 30, 100, and 300 μg/mL MKE, respectively (** p < 0.01) (Figure 4). In groups treated with 30, 100, and 300 μg/mL MKE, mPGES-1 levels were reduced by 61.28 (±1.58)%, 86.26 (±0.54)%, and 81.22 (±0.57)%, respectively, and IL-1β levels were reduced by 24.33 (±0.73)%, 38.45 (±0.85)%, and 54.38 (±0.26)% (** p < 0.01).

**Figure 5:**
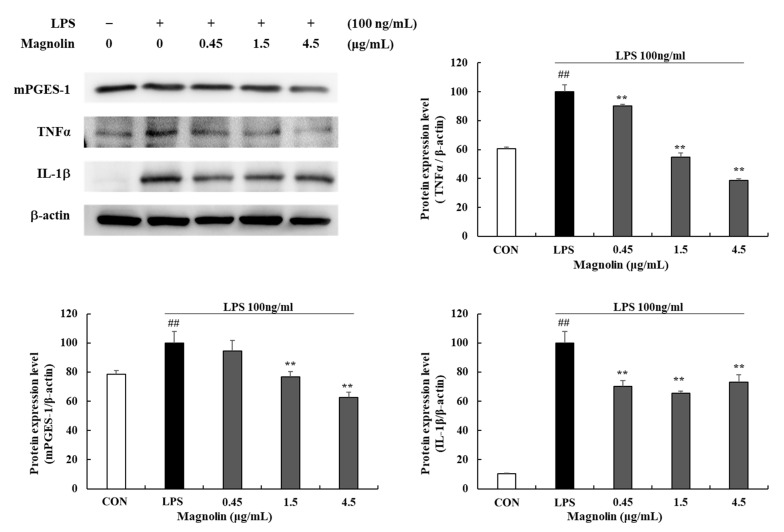
Effects of magnolin (0.45, 1.5, and 4.5 µg/mL) on the production of Inflammatory mediators in LPS-stimulated RAW 264.7 cells. Magnolin and 100 ng/mL of LPS were added, but not to the vehicle control group, and incubated at 37 °C for 24 h. Protein expression levels of TNF-α, IL-1β and mPGES-1 were detected by Western blot. The protein band density was measured utilizing ImageJ software. β-actin was used as a control. Values represent means ± standard deviation. *## p* < 0.01 vs. Vehicle control group; ** p < 0.01 vs. LPS-activated control group.

In groups treated with 0.45, 1.5, and 4.5 μg/mL magnolin, TNF-α expression levels were reduced by 9.95 (±1.38)%, 45.21 (±2.75)%, and 61.12 (±0.84)%, respectively, and IL-1β expression levels were decreased by 29.76 (±4.11)%, 34.62 (±1.68)%, and 26.73 (±4.99)%, respectively (** p < 0.01) (Figure 5). Furthermore, in cells treated with 1.5 and 4.5 µg/mL magnolin, mPGES-1 protein expression levels were decreased by 23.04 (±3.55)% and 37.55 (±3.96)%, respectively (** p < 0.01). These results indicate that magnolin might inhibit inflammatory mediators as a biologically active compound in MKE. MKE and magnolin did not decrease cell viability at the concentrations tested, indicating that their inhibitory effects on inflammatory mediator production were not related to their cytotoxicity.

### 3.4. MKE/Magnolin Inhibits Matrix Metalloproteinases (MMPs)

**Figure 6:**
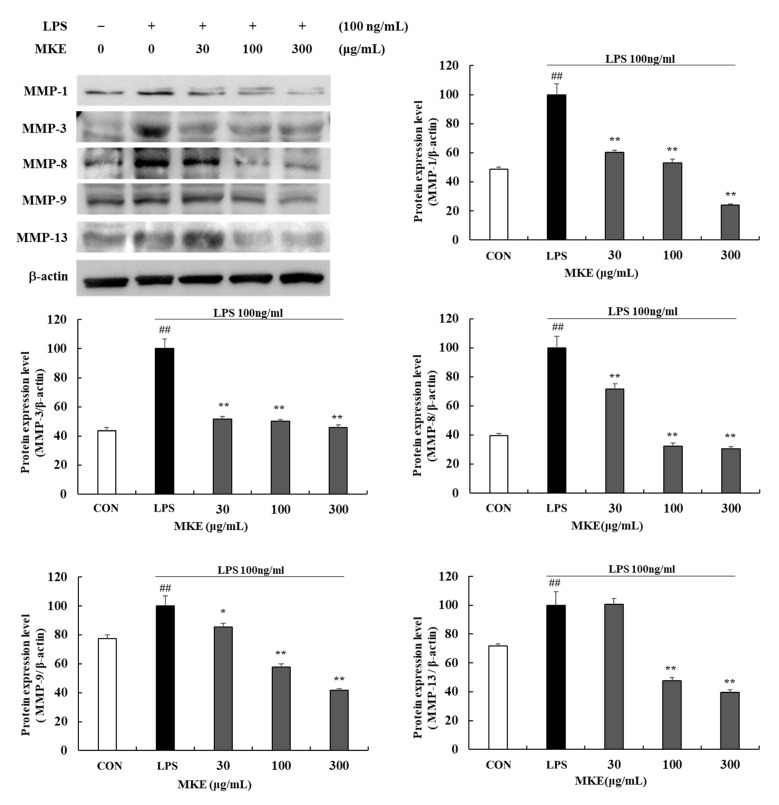
Effects of MKE (30, 100, and 300 µg/mL) on the production of MMPs in LPS-stimulated RAW 264.7 cells. MKE and 100 ng/mL of LPS was added except for in the vehicle control group and incubated at 37 °C for 24 h. Protein expression levels of MMP-1, MMP-3, MMP-8, MMP-9 and MMP-13 were detected by Western blot. The protein band density was measured by utilizing ImageJ software. β-actin was used as a control. Values represent means ± standard deviation. *## p* < 0.01 vs. Vehicle control group; * p < 0.05 and ** p < 0.01 vs. LPS-activated control group.

**Figure 7:**
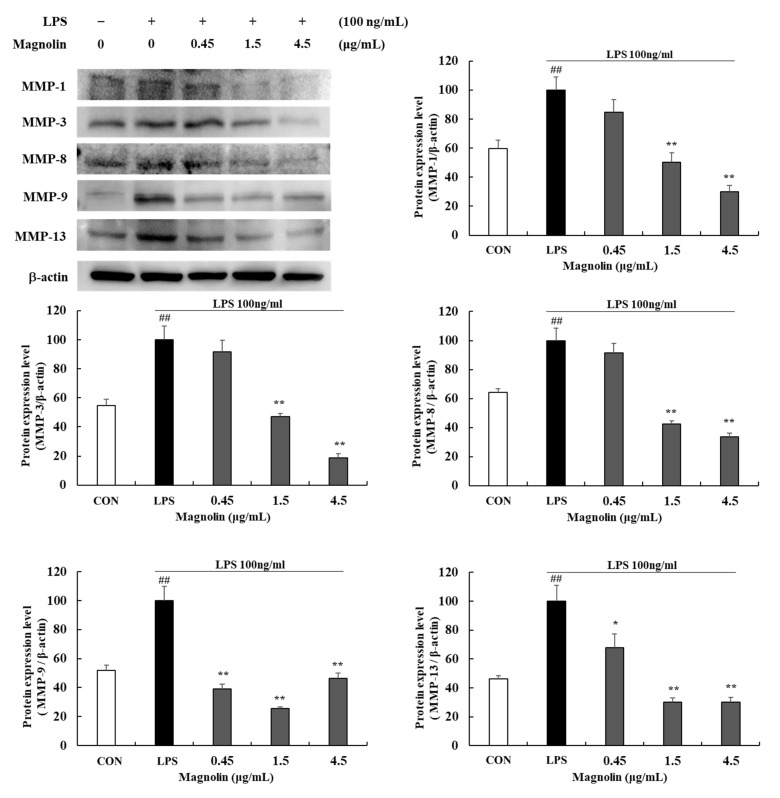
Effects of magnolin (0.45, 1.5, and 4.5 µg/mL) on the production of MMPs in LPS-stimulated RAW 264.7 cells. Magnolin and 100 ng/mL of LPS was added except for in vehicle control group and incubated at 37 °C for 24 h. Protein expression levels of MMP-1, MMP-3, MMP-8, MMP-9 and MMP-13 were detected by Western blot. The protein band density was measured by utilizing ImageJ software. β-actin was used as a control. Values represented means ± standard deviation. *## p* < 0.01 vs. Vehicle control group; * p < 0.05 and ** p < 0.01 vs. LPS-activated control group.

Treatment with MKE reduced MMP-1, -3, -8, -9, and 13 protein expression levels (Figure 6). At a concentration of 300 μg/mL, MKE reduced MMP-1, -3, -8, -9, and -13 protein expression levels by 76.14 (±1.03)%, 54.40 (±1.90)%, 69.54 (±1.40)%, 58.39 (±1.29)%, and 60.49 (±1.99)% respectively (** *p* < 0.01) (Figure 6). Magnolin treatment also significantly lowered MMP protein expression levels compared to untreated control. At 4.5 μg/mL, magnolin treatment inhibited (** p < 0.01) MMP-1, -3, -8, -9, and -13 levels by about 70.18 (±4.55)%, 81.25 (±2.85)%, 66.28 (±2.37)%, 53.57 (±3.57)%, and 69.86 (±3.16)%, respectively (Figure 7). This suggests that MKE might have potential to treat periodontal disease by inhibiting collagenases (MMP-1, -8, -13), gelatinase (MMP-9), and stromelysin (MMP-3).

### 3.5. MKE/Magnolin Inhibits TLR4/NF-κB Signaling Pathways

**Figure 8:**
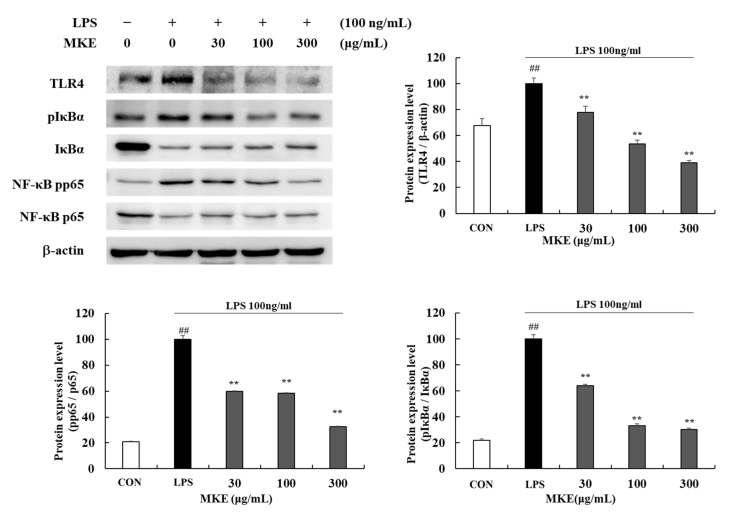
Effects of MKE (30, 100, and 300 µg/mL) on the production of TLR4 and NF-κB factors in LPS-stimulated RAW 264.7 cells. MKE and 100 ng/mL of LPS was added except for in vehicle control group and incubated at 37 °C for 15 min. Protein expression levels of TLR4, pIκBα, IκBα, pp65 and p65 were detected by Western blot. The protein band density was measured by utilizing ImageJ software. β-actin was used as a control for TLR4 protein expression level. Values represent means ± standard deviation. *## p* < 0.01 vs. Vehicle control group; ** p < 0.01 vs. LPS-activated control group.

Expression level of toll-like receptor 4 (TLR4) and phosphorylation levels of p65 and IκBα were increased in LPS-induced RAW 264.7 cells (## *p* < 0.01). Treatment with MKE at 30, 100, and 300 μg/mL reduced TLR4 by 22.28 (±4.98)%, 46.61 (±2.89)%, and 60.80 (±1.82)%, respectively (** *p* < 0.01). Furthermore, in cells treated with 30, 100, and 300 μg/mL MKE, pp65/p65 ratios were inhibited by 39.95 (±0.22)%, 41.55 (±0.42)%, and 67.42 (±0.30)%, respectively, and pIκBα /IκBα ratios were inhibited by 35.74 (±0.76)%, 66.90 (±1.43)% and 69.76 (±1.11)%, respectively (** *p* < 0.01) (Figure 8).

**Figure 9:**
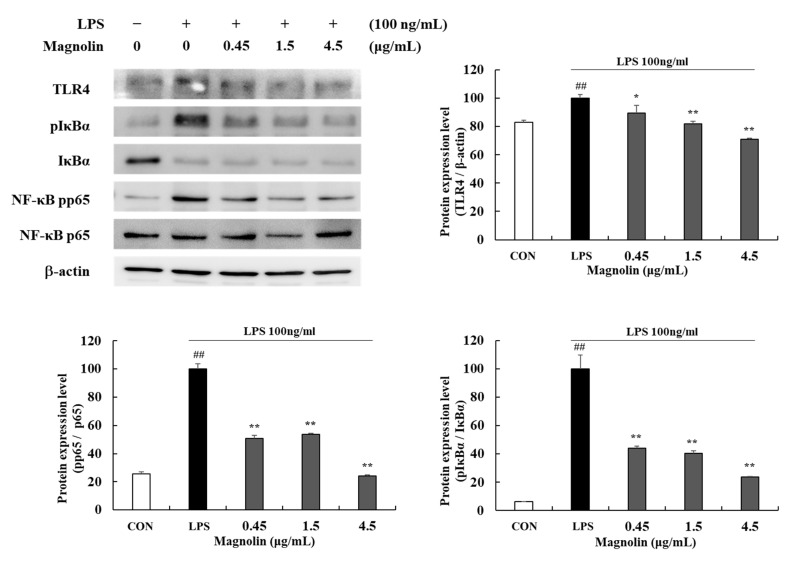
Effects of magnolin (0.45, 1.5, and 4.5 µg/mL) on the production of TLR4 and NF-κB factors in LPS-stimulated RAW 264.7 cells. Magnolin and 100 ng/mL of LPS was added except for in the vehicle control group and incubated at 37 °C for 15 min. Protein expression levels of TLR4, pIκBα, IκBα, pp65 and p65 were detected by Western blot. The protein band density was measured by utilizing ImageJ software. β-actin was used as a control for TLR4 protein expression level. Values represent means ± standard deviation. *## p* < 0.01 vs. Vehicle control group; * p < 0.05 and ** p < 0.01 vs. LPS-activated control group.

At concentrations of 0.45, 1.5, and 4.5 µg/mL, magnolin reduced TLR4 protein expression levels by 10.63 (±5.60)%, 18.01 (±1.80)%, and 29.17(±0.70)%, respectively (* *p* < 0.05, ** *p* < 0.01) (Figure 9). In addition, in cells treated with 0.45, 1.5, and 4.5 µg/mL magnolin, pp65/p65 and ratios were inhibited by 49.07 (±1.89)%, 46.33 (±0.75)%, and 76.04 (±1.00)%, respectively, and pIκBα /IκBα ratios were inhibited by 55.76 (±1.39)%, 59.74 (±1.86)%, and 76.43 (±0.60)%, respectively (** *p* < 0.01).

### 3.6. MKE/Magnolin Inhibits TAK1/MAPK Signaling Pathways

**Figure 10:**
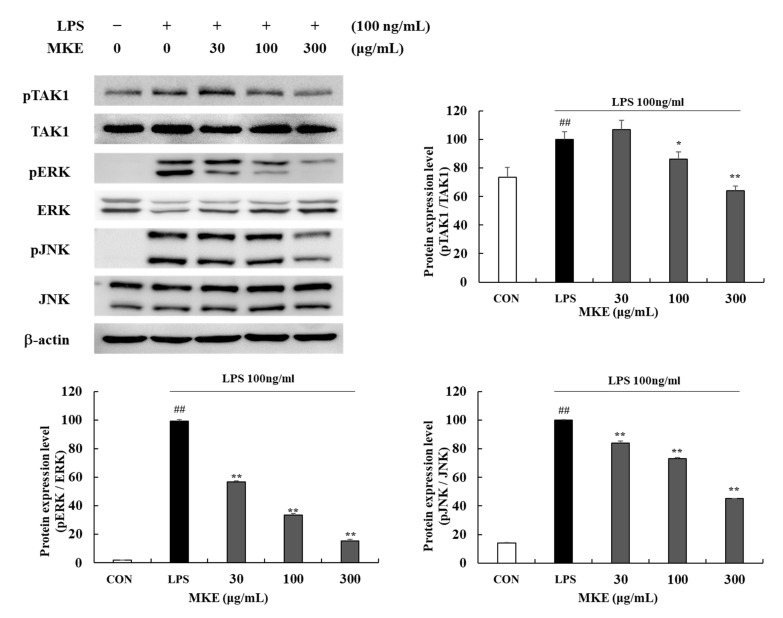
Effects of MKE (30, 100, and 300 µg/mL) on the production of pTAK1 and MAPK factors in LPS-stimulated RAW 264.7 cells. MKE and 100 ng/mL of LPS was added except for in the vehicle control group and incubated at 37 °C for 15 min. Protein expression levels of pTAK1, TAK1, pERK, ERK, pJNK, JNK were detected by Western blot. Values represent as means ± standard deviation. *## p* < 0.01 vs. Vehicle control group; * p < 0.05 and ** p < 0.01 vs. LPS-activated control group.

LPS-induced RAW 264.7 cells significantly increased (## *p* < 0.01) phosphorylation levels of TAK1 and MAPK factors c-Jun N-terminal kinase (JNK) and extracellular signal-regulated kinase (ERK). MKE at 100 and 300 μg/mL inhibited (* *p* < 0.05, ** *p* < 0.01) phosphorylation levels of pTAK1 by 13.64 (±5.05)% and 36.04 (±3.34)%, respectively. In cells treated with 30, 100, and 300 MKE μg/mL, decreases in the phosphorylation ratio of MAPK factors were by 43.23 (±0.72)%, 66.68 (±1.21)%, and 84.80 (±1.03)%, respectively, for ERK and 16.06 (±1.45)%, 27.00 (±0.71)%, and 54.85 (±0.15)%, respectively, for JNK (Figure 10).

**Figure 11:**
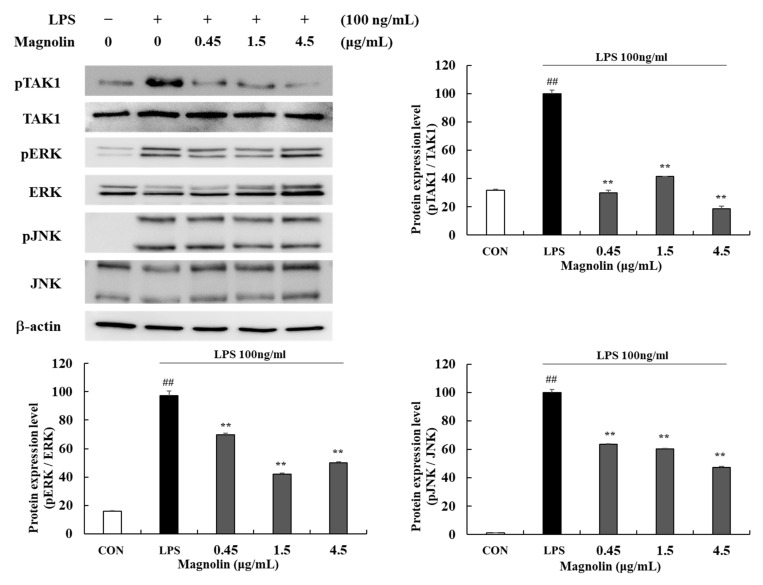
Effects of magnolin (0.45, 1.5, and 4.5 µg/mL) on the production of pTAK1 and MAPK factors in LPS-stimulated RAW 264.7 cells. Magnolin and 100 ng/mL of LPS was added except for in the vehicle control group and incubated at 37 °C for 15 min. Protein expression levels of pTAK1, TAK1, pERK, ERK, pJNK, JNK were detected by Western blot. Values represent means ± standard deviation. *## p* < 0.01 vs. Vehicle control group; ** p < 0.01 vs. LPS-activated control group.

Magnolin at 0.45, 1.5, and 4.5 μg/mL significantly (** p < 0.01) reduced phosphorylation levels of pTAK1 by 70.06 (±1.77)%, 58.61 (±0.18)%, and 81.47 (±2.03)%, respectively. In groups treated with 0.45, 1.5, and 4.5 μg/mL magnolin, phosphorylation ratios of MAPK factors were decreased by 30.47 (±1.35)%, 57.85 (±0.74)%, and 50.05 (±0.90)%, respectively, for ERK and 36.50 (±0.51)%, 39.58 (±0.30)%, and 52.55 (±0.63)%, respectively, for JNK (Figure 11). In this study, we confirmed that MKE/magnolin could inhibit TAK1 phosphorylation and MAPK activation through TLR4 regulation.

## 4. Discussion

This study was performed to evaluate the potential of MKE and magnolin for the treatment of periodontal diseases. Periodontitis is caused by inflammation induced by pathogenic oral bacteria, leading to tissue damage and clinical attachment loss [2]. In this study, we suggested the potential of MKE and magnolin for treating periodontitis through an anti-inflammatory mechanism in *P. gingivalis*-LPS activated RAW 264.7 macrophages.

*P. gingivalis* is an anaerobic, Gram-negative bacterium known to generate a variety of toxic factors that can either cause tissue destruction on their own or act through other mediators to cause inflammation [34]. The endotoxin LPS released by bacteria can trigger an inflammatory response that begins with recognition by Toll-like receptors (TLRs) on the cell surface, with production of NO, an inflammatory mediator, in macrophages [35,36]. Before conducting an NO production test, cell viability was measured by the MTT assay to determine the cytotoxicity of MKE (Figure 2). RAW 264.7 cells did not show apoptosis after treatment with 300 μg/mL MKE or 4.5 μg/mL magnolin. *P. gingivalis* LPS at 100 ng/mL increased the amount of NO production by about two to five times in RAW 264.7 cells. However, treatment with MKE at 300 μg/mL or magnolin at 4.5 μg/mL reduced the amount of NO production by 60% and 32%, respectively (Figure 3).

LPS can initiate the recognition of TLR4 and induce the interaction of adapter molecule-transforming growth factor beta-activated kinase 1 (TAK1) to activate NF-κB and MAPK signaling pathways, resulting in the production of inflammatory mediators [37]. It is known that TAK1 and NF-κB activator-binding kinase 1 signals are turned on following TLR4 activation [38]. TAK1 activated through phosphorylation can activate inflammatory mediators by regulating cell signaling through NF-κB and mitogen-activated protein kinase (MAPK) cascades [39]. Therefore, we investigated the inhibitory effect of MKE on the TAK1/MAPK pathway following activation of TLR4 by LPS treatment. We confirmed that MKE suppressed the NF-κB pathway in LPS-induced RAW 264.7 cells by reducing TLR4 protein expression and phosphorylation of p65 and IκBα. It has been reported that phosphorylation levels of MAPK-related factors are important signals associated with inflammation and periodontitis [40,41]. In this study, the ratios of p-JNK to JNK and p-ERK to ERK were increased in LPS-treated group. However, these ratios were suppressed by MKE or magnolin treatment. The phosphorylation level of TAK1 was decreased by MKE or magnolin treatment. This result suggests that MKE could inhibit periodontal-related inflammation through TLR4–TAK1–MAPK signaling.

NF-κB is known as a multi-unit transcription factor that plays an important role in proinflammatory cytokine gene induction. In the present study, we confirmed that expression levels of inflammatory cytokines such as IL-1β, TNF-α, and mPGES-1 were increased as phosphorylation levels of NF- κB and MAPK factors increased in RAW 264.7 cells. TNF-α and IL-1β are known to be involved in the induction of bone resorption and inhibition of bone formation. High levels of TNF-α and IL-1β have been observed in diseased gum tissues [42,43,44,45]. Among PGES, mPGES-1 is an enzyme induced by the stimulation of inflammatory mediators such as IL-1β and TNF-α, mainly released by macrophages after tissue damage or bacterial infection [46,47,48]. Therefore, inhibition of inflammatory cytokine expression may play an important role in preventing the progression of periodontal diseases. In this study, we demonstrated that MKE/magnolin treatment could suppress LPS-induced NF-κB activation and expression levels of TNF-α, IL-1β, and mPGES-1.

*P. gingivalis*-induced overexpression of TNF-α and IL-1β can additionally generate other inflammatory mediators such as MMPs [49]. Levels of MMPs play a key role in inflammatory processes that can lead to connective tissue loss and alveolar bone loss in periodontal disease [50]. Excessive production of MMP-1 can accelerate matrix degradation in periodontitis [51,52,53]. According to a previous study, protein expression levels of MMP-1, -8, and -13 are higher in periodontitis patients than in normal controls [54,55,56,57,58]. In chronic periodontitis patients, MMP-9 can readily digest denatured collagen, gelatin, and other extracellular matrix elements. MMP-1 and MMP-3 can act as modulators of MMP-9 [59,60]. Results of previous studies have shown that levels of MMPs are correlated with the severity of periodontitis. As a result of the present study, LPS significantly increased the expression levels of MMP-1, -3, -8, -9, and -13 proteins in RAW 264.7 cells. Treatment with MKE or magnolin decreased the expression levels of these MMPs. Therefore, MKE might contribute to the alleviation of gingival tissue destruction and periodontitis progression.

In conclusion, our study confirmed the anti-inflammatory and MMP-expression-inhibiting effects of MKE on LPS-induced RAW 264.7 cells by inhibiting TLR4–TAK1-mediated activation of NF-κB and MAPK signaling pathways. Magnolin also demonstrated effects similar to MKE, demonstrating that magnolin could act as an active ingredient in MKE. The results of this study suggest that MKE can be used as a therapeutic agent for periodontitis through its anti-inflammatory and MMP-inhibiting effects, and revealed the inhibitory mechanism of MKE against oral-disease-causing microbial endotoxins. However, this study has limitations in that it used an in vitro model, and therefore, it is necessary to evaluate the anti-periodontitis effect of MKE in animal models. Additionally, the efficacy and safety of oral administration of MKE in patients with periodontitis will be evaluated.

Through these additional studies, MKE can be developed as a botanical drug and health functional food for the prevention of periodontal disease, or the treatment of patients with periodontal diseases.

## Data Availability

Not applicable.
